# Automated Detection of Ischemic Stroke and Subsequent Patient Triage in Routinely Acquired Head CT

**DOI:** 10.1007/s00062-021-01081-7

**Published:** 2021-08-31

**Authors:** Tom Finck, David Schinz, Lioba Grundl, Rami Eisawy, Mehmet Yiğitsoy, Julia Moosbauer, Claus Zimmer, Franz Pfister, Benedikt Wiestler

**Affiliations:** 1 Department of Diagnostic and Interventional Neuroradiology, Klinikum rechts der Isar, Technische Universität München, Ismaninger Str. 22, 81675 Munich, Germany; 2 Chair for Computer Aided Medical Procedures & Augmented Reality, Technische Universität München, Munich, Germany; 3 Deepc GmbH, Munich, Germany

**Keywords:** Machine learning, Stroke, Artificial intelligence, Emergency imaging, Computed tomography

## Abstract

**Purpose:**

Advanced machine-learning (ML) techniques can potentially detect the entire spectrum of pathology through deviations from a learned norm.

We investigated the utility of a weakly supervised ML tool to detect characteristic findings related to ischemic stroke in head CT and provide subsequent patient triage.

**Methods:**

Patients having undergone non-enhanced head CT at a tertiary care hospital in April 2020 with either no anomalies, subacute or chronic ischemia, lacunar infarcts of the deep white matter or hyperdense vessel signs were retrospectively analyzed. Anomaly detection was performed using a weakly supervised ML classifier. Findings were displayed on a voxel-level (heatmap) and pooled to an anomaly score. Thresholds for this score classified patients into i) normal, ii) inconclusive, iii) pathological. Expert-validated radiological reports were considered as ground truth. Test assessment was performed with ROC analysis; inconclusive results were pooled to pathological predictions for accuracy measurements.

**Results:**

During the investigation period 208 patients were referred for head CT of which 111 could be included. Definite ratings into normal/pathological were feasible in 77 (69.4%) patients. Based on anomaly scores, the AUC to differentiate normal from pathological scans was 0.98 (95% CI 0.97–1.00). The sensitivity, specificity, positive and negative predictive values were 100%, 40.6%, 80.6% and 100%, respectively.

**Conclusion:**

Our study demonstrates the potential of a weakly supervised anomaly-detection tool to detect stroke findings in head CT. Definite classification into normal/pathological was made with high accuracy in > 2/3 of patients. Anomaly heatmaps further provide guidance towards pathologies, also in cases with inconclusive ratings.

## Introduction

Computed tomography (CT) of the head remains the primary modality in stroke imaging. The CT appearances of brain ischemia vary considerably from obvious defects in chronic ischemia to somewhat less obvious findings such as hyperdense vessel sign (HVS) as a surrogate for occluding thrombus. Thus, beyond the mere exclusion of hemorrhagic stroke, the time-critical detection of CT findings associated with ischemic stroke underlines how timely and qualified interpretation of CT scans is a cornerstone of adequate patient management.

Continuing advances in the machine-learning (ML) domain are promising to relieve the conflict between rising examination counts and finite human resources such as reading and interpretation time. In general, ML tools can learn through supervised or unsupervised training, with the latter not needing explicit labels for each of the classes (i.e. pathologies) it is supposed to detect. Most ML approaches in medical imaging are however based on strongly supervised learning with the associated need for labor-intensive pixel-wise image segmentation and the inherent limitation that a system can only detect what it has previously “seen”. Unsupervised or weakly supervised (only requiring global class labels instead of pixel-level segmentations) systems, on the other hand, offer the potential to learn the underlying data distribution and thus flag pathology if a derivation from this learned norm is found. In doing so, pathology is defined as a deviation from an internalized normal reference and the whole spectrum, not just a predefined library, of imaging anomalies could potentially be detected.

We have developed and evaluated a weakly supervised anomaly detection system based on this principle of learning normal anatomy in head CT and flagging anomalies linked to ischemic stroke as deviations from this norm. Here we report first data on the performance of this system in detecting the wide variety of ischemic stroke-related CT findings and provide evidence for patient triage based on this system.

## Methods

### Dataset

This retrospective analysis of a single tertiary care center was approved by the local IRB, and the need for informed consent was waived. To avoid selection bias, all patients referred to the neuroradiology department of a university hospital for non-enhanced head CT in April 2020 were considered eligible for this study. Of these, only scans showing either normal brain, chronic ischemia, subacute ischemia, lacunar deep white matter (DWM) infarcts or HVS were considered. We decided to restrict the dataset to healthy and stroke patients only in order to make a better assessment of the disease-specific performance of the anomaly detection tool.

For HVS, the retrieving period was extended to January 2020–April 2020 as this image finding without concomitant anomalies was only rarely encountered. Only one scan/patient was included and scans depicting > 1 of the above-mentioned pathologies or concurring pathologies were excluded to prevent class overlap. The same hardware (Philips Ingenuity 5000, Philips Medical Systems, Best, The Netherlands) was used in all patients with local postprocessing according to a manufacturer-specific iterative model reconstruction (IMR3).

### Data Processing and System Architecture

Anatomical correspondence of DICOM images to an internal atlas was established through image registration using the Advanced Normalization Tools (ANTs) framework where rigid, affine and deformable alignments were used to co-register every image to the template image of the internal atlas [1]. The anomaly detection model was trained using a weakly supervised machine learning strategy, which only required a small, weakly annotated dataset. Importantly, no pixel-level annotations (i.e. segmentations) were necessary for this method. First, a (multivariate) density was estimated over the anatomical regions of a diverse cohort of 191 normal scans from multivendor scanners (co-registered to the above-mentioned template) used for training the algorithm. Per-voxel Gaussian density distributions were fitted across the co-registered training dataset. Finally, per-voxel upper and lower bounds of the 90% confidence intervals were calculated. Outlier voxels in test scans were identified by comparing against the voxel-wise upper and lower bounds of the confidence interval in the internal atlas.

Based on this estimated distribution, deviations from the spatial distribution of HU densities were inferred and summarized by an anomaly score ranging from 0 to 1. This anomaly score is converted into classes (“normal”, “pathological”, “inconclusive”) via thresholding. The thresholds are scanner-specific and were calibrated on an independent, mixed validation dataset (not used in this study) of 61 anonymized scans (globally labelled as “normal” or “pathological”) from the local CT scanner to minimize the false positive rate under the constraint of a false omission rate of 0. Pathologies in the validation set were not restricted to stroke-specific findings but further included various findings, such as intracranial bleeding or masses.

In cases where the anomaly score was above a threshold (calibrated with the in-house data as stated), the anomalous finding was added to a heat map and the patient was labelled as “pathological”. Anomaly scores below a second threshold led to a label of “normal”, while anomaly scores between the upper and lower threshold led to an “inconclusive” rating.

At last, both the anomaly map showing the estimated spatial deviations and patient-level prediction into normal/pathological/inconclusive were displayed via a browser-based user interface for review. On selection of a patient, the scan with the anomaly detection overlaid as a heat map was shown to the radiologist.

### Ground Truth Assessment

The accuracy of the systems predictions was judged against the radiological report, cosigned by at least two neuroradiologists during clinical routine. Nomenclature of pathology labels in the radiological reports corresponded to established criteria: subacute ischemia was defined as ischemic demarcation of a vascular territory in head CT with concomitant symptom onset 24 h–1 week prior to the scan; chronic ischemia was defined as brain parenchyma hypodensity within a vascular territory with concomitant symptom onset > 3 weeks prior to imaging [2]; lacunar DWM infarcts were defined as defects in the DWM measuring > 15 mm in diameter [3] and HVS was present if attenuation within a proximal brain-supplying vessel was > 1.2 times that of the contralateral artery [4].

The individual heat map for each patient was compared to the written report as well as the original DICOM images in order to assess if anomalies had reliably been detected or if false positive detections were made.

Normal scans correctly identified as such were defined as true negative, while correct segmentation of a pathology with subsequent labelling of the patient as pathological was defined as true positive. Analogously, normal scans labelled as pathological were false positive and scans showing a pathology wrongfully labelled as normal were considered false negative. In scans with inconclusive ratings and pathological findings on ground truth, segmentations were analyzed to verify whether the pathology in question had been detected or had been missed.

### Statistical Analysis

The diagnostic accuracy to discriminate between normal and pathological head CTs was chosen as the primary endpoint and assessed through calculation of the area under the receiver operator curve (AUC) based on the respective anomaly scores, as described before [5]. To determine the maximum potential effectiveness of the system, the Youden index was calculated based on this AUC analysis [6].

Sensitivities, specificities, positive predictive values (PPV), and negative predictive values (NPV) were further calculated based on the patient labels given by the algorithm and corresponding ground truth. To prevent inflation of performance metrics and in line with the rationale of a screening test, inconclusive labels were considered either true positive (if disease was present on ground truth and correctly detected by the voxel-wise segmentations) or false positive (if disease was not present on ground truth) [7]. Statistical analysis was performed using Graphpad Prism Version 8.4.3 (Graphpad Software, San Diego, CA, USA). *P*-values below 0.05 were considered statistically significant.

## Results

### Study Cohort and Image Processing

Of 340 CT scans from 208 patients that were acquired during the retrieving period, 111 scans (1 scan/patient; mean age 66.1 ± 19.9 years, 56.7% male) could be included for analysis.

Of these 32 were normal according to the ground truth report and 79 showed stroke-related anomalies (HVS in 28 patients, subacute ischemia in 22 patients, chronic ischemia in 22 patients and lacunar DWM infarcts in 7 patients).

**Fig. 1:**
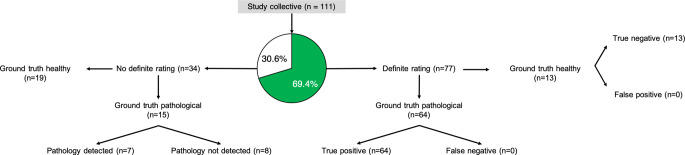
Flow-chart illustrating the triage performance of the algorithm. Definite ratings were given in 77/111 (69.4%) patients with no false-positives or false-negatives in the case of definite ratings

Definite patient-level ratings into normal or pathological were provided in 77/111 scans, translating to a test yield of 69.4%. Consequently, inconclusive ratings were given in 34/111 (30.6%) patients, of whom 15 (44.1%) had pathological findings in ground truth and 19 (55.9%) had normal scans (Fig. 1). In inconclusive ratings, the leading pathology has been missed (i.e. not included in the voxel-level anomaly segmentations) in 8/15 (53%) patients (as shown in Figs. 1 and 3). Notably, definite ratings were given significantly more often in pathological (81.0%) than normal (40.6%) scans (*p* < 0.0001) (Table 1).

**Table 1:** Given are the numbers of patients according to their labels with the respective share of definitely labelled (normal/pathological) and inconclusively labelled scans for each class

Ground Truth	All (*n* = 111)	Definite rating (*n* = 77)	Inconclusive rating (*n* = 34)	*p* for difference	% of definite ratings
Normal	32	13	19	0.14	40.6
Ischemia—chronic	22	20	2	< 0.0001	90.9
Ischemia—subacute	22	20	2	< 0.0001	90.9
HVS	28	19	9	0.002	67.9
Lacunary DWM infarct	7	5	2	0.13	71.4
All pathological	79	64	15	< 0.0001	81.0

*HVS* hyperdense vessel sign, *DWM* deep white matter

### Performance Metrics

Based on the calculated anomaly scores, the diagnostic accuracy to dichotomize between normal and pathological CT scans was excellent with an area under the ROC of 0.98 (95% CI 0.97–1.00). As classification by the algorithm is threshold-based, considerable differences could be noted in the anomaly scores of normal 0.257 ± 0.249 vs. pathological 0.912 ± 0.157 head CTs (*p* for difference < 0.0001). In scans with stroke findings, anomaly scores ranged from 0.966 ± 0.07 in chronic ischemia to 0.878 ± 0.215 in HVS. Table 2 provides detailed information on diagnostic accuracy and anomaly scores.

**Table 2:** Given is the diagnostic accuracy to discern normal scans from scans with stroke findings and subgroups thereof

	AUC (95% CI)	Anomaly score
Normal	–	0.257 ± 0.249
All pathological	0.979 (0.968–1.00)	0.912 ± 0.157^a^
Ischemia—chronic	0.997 (0.989–1.00)	0.966 ± 0.07^a^
Ischemia—subacute	0.986 (0.957–1.00)	0.934 ± 0.120^a^
HVS	0.981 (0.952–1.00)	0.878 ± 0.215^a^
Lacunar DWM infarct	0.991 (0.968–1.00)	0.899 ± 0.124^a^

*HVS* hyperdense vessel sign, *DWM* deep white matter, *AUC* Area under the curve, *CI* Confidence interval

^a^Significantly different from the anomaly scores of normal scans

In an effort to realistically reflect the utility of the triage tool presented here, scans with inconclusive ratings were considered either true positive (if disease was present on ground truth) or false positive (if disease was absent on ground truth), as stated in the methods. In doing so, the sensitivity, specificity, positive predictive value and negative predictive value was 100% ((64 + 15)/(64 + 15 + 0)*100), 40.6% (13/(13 + 0 + 19)*100), 80.6% ((64 + 15)/(64 + 15 + 19)*100) and 100% (13/(13 + 0)*100), respectively (input data for calculating the respective metrics are provided in Table 3).

**Table 3:** Given are the metrics used for calculating the diagnostic accuracy in the study cohort

	Ground truth	
Algorithm output	Stroke findings present	Stroke findings absent
Pathological	64	0
Inconclusive	15	19
Normal	0	13

In the subgroup analysis for the 77 scans with definite ratings, categorization into true positive, false positive, true negative and false negative was done for 64, 0, 13 and 0 patients, respectively. This translated to a sensitivity and specificity of 100% each. As there was neither a scan with stroke-related anomalies wrongfully labeled as normal nor a normal scan wrongfully labeled as pathological, the negative and positive predictive values in the case of definite ratings were both 100%.

Based on the ROC derived from the anomaly scores, an optimal Youden index of 0.92 was found for an anomaly score (upper cut-off) of 0.775. For the entire cohort, this post hoc determination led to theoretical sensitivity and specificity of 92.3% and 100%, respectively.

**Fig. 2:**
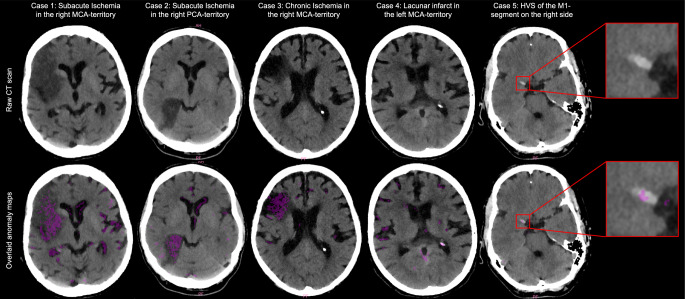
Case examples for correct detection and patient labeling in subacute ischemia (cases 1 and 2), chronic ischemia (Case 3), lacunar DWM infarcts (Case 4) and HVS (Case 5). Shown are the raw CT scans (*top panel*) with the corresponding anomaly maps (*lower panel*, *segmentations of anomalous findings in pink*). *DWM* deep white matter, *HVS* hyperdense vessel sign, *MCA* middle cerebral artery, *PCA* posterior cerebral artery

**Fig. 3:**
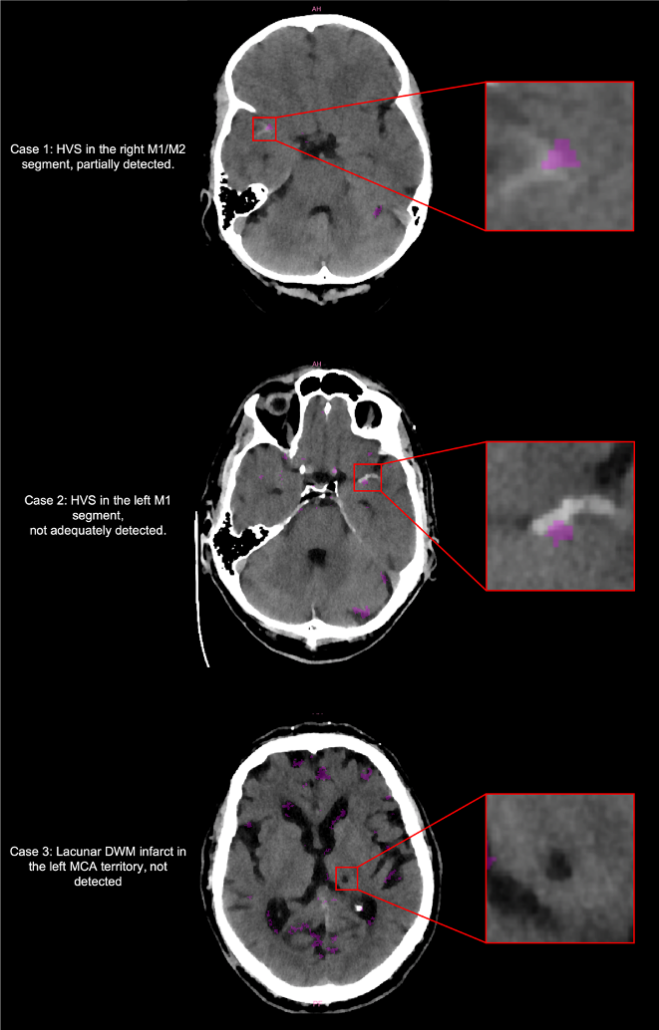
Case examples for inconclusive ratings. Cases 2 (HVS) and 3 (Thalamic Infarct) show scans where the underlying pathology was not adequately detected in the anomaly maps (*segmentations of anomalous findings in pink*). Case 1 shows partial detection of the underlying HVS that did not reach the threshold for correct patient categorization as pathological. *DWM* deep white matter, *HVS* hyperdense vessel sign, *MCA* middle cerebral artery

To illustrate the strengths and shortcomings of the system we provide exemplary cases for correctly and inconclusively classified patients in Figs. 2 and 3.

## Discussion

Ischemic stroke continues to be a leading cause of morbidity and mortality worldwide [8]. As recommended by societal guidelines, head CT remains the primary workhorse in neuroradiological emergency imaging [9]. Findings related to ischemic stroke cover a wide range of appearances, ranging from hypodense defect areas in chronic ischemia to hyperdensity of a brain-supplying artery on the basis of thrombotic occlusion. Missing these anomalies can have detrimental effects on patient outcome [10]. Quick detection of stroke-related findings is often essential to provide adequate treatment and, in light of the conflict between the efforts of commercial and public healthcare providers to save costs on still expensive human resources, and the rising counts of imaging studies, computer-aided diagnostics could in part relieve this conflict by providing a study list hierarchically sorted according to the degree of imaging abnormalities.

This study provides data on the utility of a weakly supervised stroke findings detection tool in a continuous series. Definite predictions on the presence/absence of stroke-related findings could be made in ≈ 70% of individuals after a processing time of roughly 1 min. This went along with an excellent diagnostic accuracy to discriminate normal scans from those with ischemic stroke and a negative predictive value of 100%.

Beyond patient-level predictions, the anomaly maps can further guide a clinician towards relevant findings and have the potential to increase the value of CT studies by combining critical human interpretation with software-based pathology flagging.

Continued threshold calibration further holds the promise to improve classification completeness and calls for future research into continuous learning approaches for this system.

Automated detection of stroke-associated findings in medical imaging is a field that has witnessed major developments in recent years and given rise to tools aimed at detecting parameters such as demarcation of ischemic parenchyma or mismatch volumes on CT perfusion maps [11–14]. In detail, automated determination of ASPECTS, a topographic scoring system that divides the MCA territory into regions of interest and quantifies ischemic damage, has even been suggested for selecting patients who could benefit most from mechanical thrombectomy [15]. First reports on the feasibility to extract the features of HVS have furthermore emerged and added to strong evidence for ML-based quantification of tissue at risk [13, 16]. Albeit impressive, the Achilles’ heel of most of these systems remains their foundation on supervised class learning. This necessitates class-specific training, potentially reducing their robustness in atypical findings.

Turning around this pathology-centered approach is promising to expand the spectrum and heterogeneity of anomalies that can be detected. Therefore, our ML tool tracks deviations from an internally acquired multidimensional reference presentation, i.e. it learns the normal anatomical variability of the human brain and defines pathology as every state that is discrepant from this reference.

Systems not requiring manual feature encoding have been implemented before but were either restricted to detecting other entities (mainly intracranial hemorrhage) or applied to a preselected patient cohort in an experimental setting that does not reflect the unfiltered clinical workflow [17, 18].

The essence of a triage system is to categorize patients based on the lack or presence of a condition and hence channel resources towards the cases needing most urgent attention. Ad initio, each patient with inconclusive results thus needs to be considered test positive before proven otherwise. We chose to follow this concept as it is most reflective of the clinical reality and prevents promotion of suboptimal test strategies through overstating summary statistics. On the other hand, this approach has highlighted the potential to improve the system’s performance if the share of inconclusive results can be reduced through continuous learning mechanisms.

A limitation of this study is that the reported accuracy measures are constrained by the Bayes theorem according to which pre-test probabilities affect post-test probabilities. This means that our study setting in a university hospital (where patients with low clinical pre-test probability for stroke are in many cases investigated with MRI) has led to relatively low counts of healthy controls and a high proportion of stroke patients. This could lead to a distortion of accuracy measures in hospitals having differing patient demographics. Also, in order to specifically assess the diagnostic accuracy to detect stroke-related findings, we limited our study cohort to patients with normal CT scans and ischemic stroke, only. One can however speculate that the architecture of our system should be able to detect simultaneously occurring intracranial pathologies as this would constitute an even wider deviation from the internalized reference atlas. Second, generalizability to centers with other hardware equipment should be explored and was not considered in this study that aimed to reflect the clinical workflow at one site. Not all imaging stigmata included in this study carry the same clinical relevance. As such, detection of subacute ischemia entails more timely work-up and secondary prophylaxis than depiction of DWM infarcts; however, we prioritized categorization of multiple ischemia-related CT findings in order to move away from single class detection systems potentially encountering weaknesses in more heterogenous datasets. It should be said, however, that hyperacute stroke (defined as 0–24 h after symptom onset, as in [2]) were not included in this study given that our institute-specific imaging protocol in such cases includes CT perfusion data and hence unbiased reports of nonenhanced head CTs were not available for this unselected retrospective series. Notable performance drops should nonetheless be expected in such cases as the mechanism of the system investigated here is based on quantitative derivations within image data, and these derivations can be very subtle in (hyper)acute stroke. This becomes evident in case 1 (Fig. 2) where only the central, most hypodense ischemic areas in the right MCA territory have been segmented on the anomaly maps while a large part of the cortical/subcortical parenchyma has not been flagged as anomalous by the algorithm. Next, the fact that a radiologist’s attention is guided towards the areas highlighted in the anomaly map could promote “satisfaction of search”. Anomaly maps should only be used as a guide towards pathology in scans flagged as pathological, as the high sensitivity of the system presented here will inevitably lead to the segmentation of a small proportion of inconspicuous voxels. This is highlighted by the anomaly score of normal CT scans that was 0.257, instead of zero as it would be in a perfectly calibrated setting. The influence of this noise in anomaly maps has been mitigated by using a threshold-based model for classification on the patient-level. Finally, the fact that scans showing pathology had a notably smaller share of inconclusive ratings when compared to normal scans underlines how the system was tuned in an effort to minimize false negative labels at the cost of reducing the share of definite predictions. While this undeniably leads to second look verifications of indeterminately labeled scans, accepting a relevant share of false positive classifications is an inevitable compromise to not miss any pathological scans.

In conclusion, we report on the potential utility of a weakly supervised anomaly detection system for identifying ischemic stroke-related CT findings in an unselected patient cohort. We noted a high accuracy to discriminate patients with subacute ischemia, chronic ischemia, lacunar DWM infarcts and HVS from healthy controls. Definite predictions into normal/pathological were given in close to 70% of scans, allowing adequate triage in an unselected dataset with conspicuous stroke-related findings. Through provision of patient-level labels and detailed anomaly segmentations, clinicians could in the future be provided with study lists that hierarchically sort patients according to the degree of imaging abnormalities.

## Abbreviations

ANTs: Advanced normalization tools
ASPECTS: Alberta stroke program early CT score
AUC: Area under the curve
CT: Computed tomography
DWM: Deep white matter
HVS: Hyperdense vessel sign
ML: Machine learning
MRI: Magnetic resonance imaging
NPV: Negative predictive value
PPV: Positive predictive value
ROC: Receiver operator characteristics
